# Ancestry-specific associations identified in genome-wide combined-phenotype study of red blood cell traits emphasize benefits of diversity in genomics

**DOI:** 10.1186/s12864-020-6626-9

**Published:** 2020-03-14

**Authors:** Chani J. Hodonsky, Antoine R. Baldassari, Stephanie A. Bien, Laura M. Raffield, Heather M. Highland, Colleen M. Sitlani, Genevieve L. Wojcik, Ran Tao, Marielisa Graff, Weihong Tang, Bharat Thyagarajan, Steve Buyske, Myriam Fornage, Lucia A. Hindorff, Yun Li, Danyu Lin, Alex P. Reiner, Kari E. North, Ruth J. F. Loos, Charles Kooperberg, Christy L. Avery

**Affiliations:** 1 University of North Carolina Gillings School of Public Health, 135 Dauer Dr, Chapel Hill, NC 27599 USA; 2 University of Virginia Center for Public Health Genomics, 1355 Lee St, Charlottesville, VA 22908 USA; 3 Fred Hutchinson Cancer Research Center, 1100 Fairview Ave N, Seattle, WA 98109 USA; 4 Department of Genetics, University of North Carolina at Chapel Hill, 120 Mason Farm Road, Chapel Hill, NC 27599 USA; 5 University of Washington, 1730 Minor Ave, Ste 1360, Seattle, WA 98101 USA; 6 Stanford University School of Medicine, 291 Campus Dr, Stanford, CA 94305 USA; 7 Vanderbilt University, 2525 West End Ave #1100, Nashville, TN 37203 USA; 8 University of Minnesota, 420 Delaware St SE, Minneapolis, MN 55455 USA; 9 Rutgers University, 683 Hoes Ln W, Piscataway, NJ 08854 USA; 10 University of Texas Houston, 7000 Fannin Street, Houston, TX 77030 USA; 11 National Human Genome Research Institute, 31 Center Dr, Bethesda, MD 20894 USA; 12 University of Washington, 1705 NE Pacific St, Seattle, WA 98195 USA; 13 Icahn School of Medicine at Mount Sinai, 1468 Madison Ave, New York, NY 10029 USA

**Keywords:** Blood cell traits, Combined-phenotype analysis, Pleiotropy, Diversity, Multi-ethnic, GWAS

## Abstract

**Background:**

Quantitative red blood cell (RBC) traits are highly polygenic clinically relevant traits, with approximately 500 reported GWAS loci. The majority of RBC trait GWAS have been performed in European- or East Asian-ancestry populations, despite evidence that rare or ancestry-specific variation contributes substantially to RBC trait heritability. Recently developed combined-phenotype methods which leverage genetic trait correlation to improve statistical power have not yet been applied to these traits. Here we leveraged correlation of seven quantitative RBC traits in performing a combined-phenotype analysis in a multi-ethnic study population.

**Results:**

We used the adaptive sum of powered scores (aSPU) test to assess combined-phenotype associations between ~ 21 million SNPs and seven RBC traits in a multi-ethnic population (maximum *n* = 67,885 participants; 24% African American, 30% Hispanic/Latino, and 43% European American; 76% female). Thirty-nine loci in our multi-ethnic population contained at least one significant association signal (*p* < 5E-9), with lead SNPs at nine loci significantly associated with three or more RBC traits. A majority of the lead SNPs were common (MAF > 5%) across all ancestral populations. Nineteen additional independent association signals were identified at seven known loci (*HFE*, *KIT*, *HBS1L/MYB*, *CITED2/FILNC1*, *ABO*, *HBA1/2*, and *PLIN4/5*). For example, the *HBA1/2* locus contained 14 conditionally independent association signals, 11 of which were previously unreported and are specific to African and Amerindian ancestries. One variant in this region was common in all ancestries, but exhibited a narrower LD block in African Americans than European Americans or Hispanics/Latinos. GTEx eQTL analysis of all independent lead SNPs yielded 31 significant associations in relevant tissues, over half of which were not at the gene immediately proximal to the lead SNP.

**Conclusion:**

This work identified seven loci containing multiple independent association signals for RBC traits using a combined-phenotype approach, which may improve discovery in genetically correlated traits. Highly complex genetic architecture at the *HBA1/2* locus was only revealed by the inclusion of African Americans and Hispanics/Latinos, underscoring the continued importance of expanding large GWAS to include ancestrally diverse populations.

## Background

In the average adult, 200 billion red blood cells (RBCs) are generated daily from hematopoietic stem cells in the bone marrow. The most commonly assessed traits for mature RBCs are hematocrit (HCT), hemoglobin concentration (HGB), mean corpuscular hemoglobin (MCH), MCH concentration (MCHC), mean corpuscular volume (MCV), RBC count (RBCC), and red cell distribution width (RDW); together, these traits are used to characterize RBC development and function, diagnose anemic disorders, and identify risk factors for complex chronic diseases [1–6]. RBC traits also are moderately to highly heritable, making these complex quantitative traits excellent candidates for genomic interrogation [7–9]. Improved characterization of RBC molecular pathways has benefitted both disease diagnosis and pharmaceutical development, as has been demonstrated by recent successes in a *BCL11A*-silencing gene therapy clinical trial for individuals with sickle cell disease (SCD) [10, 11].

Genetic association studies have reported over 500 independent loci for RBC traits [12–31]. However, several research gaps remain which may be addressed via recently developed methods and broadly representative study populations. First, previously published RBC trait genome-wide association study (GWAS) populations have mostly been ancestrally homogeneous [31–39]. Utilization of diverse study populations can improve identification of rare or ancestry-specific variants located in biological pathways that affect phenotypes in global populations and, when summary data are made publicly available, enable construction of broadly applicable polygenic risk scores [40]. Relatedly, gaps between estimated heritability and the proportion of variance explained by GWAS findings suggest that additional associations remain to be identified, including rare variants and independent secondary associations at known loci that are both more likely to be ancestrally specific [12, 41, 42]. Finally, RBC traits exhibit modest to high correlation, and several dozen loci have been reported for two or more RBC traits, although few studies have leveraged this shared genetic architecture to increase statistical power to map novel RBC trait loci [12, 20, 26, 43–45].

In this work, we examined the individual and shared genetic architecture of seven RBC traits in participants of the ancestrally diverse Population Architecture using Genomics and Epidemiology (PAGE) study [46]. Our findings reinforce the necessity of incorporating multi-ethnic study populations in genomics in order to accurately characterize RBC trait loci and encourage equitable application of the results to translational work [39]. The complexity of association signals at loci previously characterized in European- and East Asian-ancestry populations also demonstrates improved power to perform conditional analysis using a combined-phenotype model [47].

## Results

The number of participants with both phenotype and genotype data ranged from 33,549 (RDW) to 67,885 (HCT, see Methods, Tables S2 & S3). Seventy-eight percent of participants were female and participants were on average 57 years old at time of blood collection (Table S4). Self-reported race/ethnicity in the total study population was approximately 20% African American, 30% Hispanic/Latino, and 40% European American (Table S3).

### Combined-phenotype analyses

**Fig. 1:**
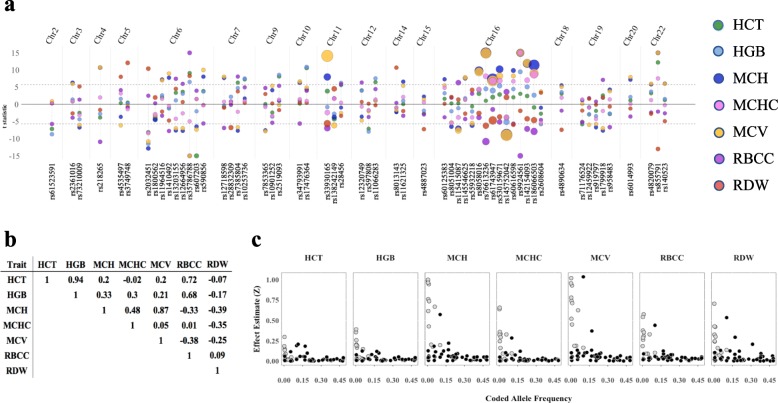
Identification and characterization of 58 independent lead variants in 39 loci in a multi-ethnic study population. **a** Lead and conditionally independent SNPs from combined-phenotype analysis of total study population show shared genetic architecture directionally consistent with correlation structure. Colored circles to the right of figure correspond to trait-specific associations. X-axis: rsid (bottom) sorted by chromosome (top) and position; y-axis: significance of association and direction of effect, represented by t-value (scaled to a maximum of t = |15|). Size of circles is exponentially proportional to effect size standardized to trait means (3^Z^) to demonstrate differences in average effect size at lead SNPs by trait. Dashed gray lines correspond to genome-wide-significance threshold of *a* = 5E-09. **b** RBC trait pair partial correlations among MEGA-genotyped participants adjusted for linear regression model covariates (*n* = 29,090 for HCT, HGB, and MCHC measurements; *n* = 22,330 for MCH, MCV, and RBCC; *n* = 19,573 for RDW). **c.** Low-frequency and rare alleles exhibit larger magnitude of effect across RBC traits in the total multi-ethnic study population. X-axis: minor allele frequency; y-axis: effect size standardized to trait mean (|Z|). Filled circles represent variants present in all ancestry sub-populations; open circles are monomorphic in one or more ancestries

Approximately 21 M SNPs met our inclusion criteria and were evaluated in our primary analysis, a combined-phenotype multi-ethnic meta-analysis of seven RBC traits. SNP associations with the combined phenotype multi-ethnic meta-analysis exceeded genome-wide significance at 39 loci (*p* < 5E-09, Figures S1, S2), all of which were identified previously. Lead SNPs at nine loci (*KIT, HFE, HBS1L/MYB, IKZF1, TFR2, HBB, HBA1/2, GCDH,* and *TMPRSS6*) were associated with three or more RBC traits at genome-wide significance (Tables 1, S5A). HCT, HGB, and MCHC exhibited genome-wide-significant associations at the fewest loci (eleven, ten, and six, respectively), whereas MCH and MCV had the most (twenty and twenty-one, respectively, Fig. 1a, Table S5A). Estimated partial correlations by RBC trait pair ranged from HCT-MCHC (partial correlation *ρ* = − 0.02) to HCT-HGB (*ρ* = 0.94, Fig. 1b). Consistent with other GWAS of quantitative complex traits, effect size was inversely correlated to allele frequency across all phenotypes (Fig. 1c).

**Table 1:** RBC trait loci with evidence of multiple independent signals among PAGE study participants

	Signal	Chr:pos	Ref/Alt	CAF^a^			*p* values									
							Multi-ethnic RBC trait-specific							Combined phenotype by race/ethnicity^a^		
				AA	HL	EU	HCT *N* = 67,885	HGB *N* = 67,870	MCH *N* = 41,317	MCHC *N* = 67,856	MCV *N* = 41,276	RBCC *N* = 41,310	RDW *N* = 33,549	AA *N* = 16,802	HL *N* = 20,697	EU *N* = 29,513
*HFE* locus																
rs2032451	1	6:26092170	T/G	0.96	0.88	0.85	4.0E-16	1.4E-30	8.2E-38	1.8E-22	2.3E-27	0.01	3.4E-25	2.0E-4	2.3E-3	1.0E-11
rs1800562	2	6:26093141	A/G	0.98	0.98	0.93	1.4E-4	2.4E-5	7.7E-30	3.1E-3	1.2E-3	0.78	0.05	1.0E-5	1.0E-10	1.0E-11
*CCND3* locus																
rs1410492	1	6:41907855	C/G	0.94	0.86	0.75	0.24	0.21	1.0E-14	0.59	3.5E-19	1.5E-14	1.9E-6	0.04	1.4E-7	1.0E-11
rs11964516	2	6:41860252	T/C	0.15	0.16	0.17	0.04	0.01	5.6E-13	0.12	3.0E-12	8.8E-4	0.03	0.02	1.6E-6	1.0E-11
*HBS1L/MYB* locus																
rs35786788	1	6:135419042	A/G	0.92	0.85	0.74	8.8E-20	7.0E-11	3.0E-66	6.2E-07	1.1E-59	3.3E-60	1.6E-16	8.0E-7	1.0E-11	1.0E-11
rs12664956	2	6:135384188	T/C	0.22	0.26	0.37	2.6E-4	2.3E-3	1.1E-14	7.9E-3	2.4E-12	2.0E-9	0.52	0.03	8.1E-5	1.0E-6
*CITED2* locus																
rs590856	1	6:139844429	A/G	0.63	0.41	0.45	1.2E-4	2.1E-4	7.7E-16	0.44	1.4E-23	2.2E-8	0.79	1.3E-4	1E-11	5.1E-7
rs607203	2	6:139841653	T/C	0.79	0.93	0.96	0.02	0.04	7.9E-11	0.12	2.1E-13	8.8E-5	0.13	5.3E-3	6.2E-6	2.9E-7
*ABO* locus																
rs2519093	1	9:136141870	T/C	0.89	0.85	0.80	5.7E-16	8.7E-18	0.32	0.05	0.85	1.1E-7	0.09	2.1E-4	1.1E-8	1.0E-8
rs10901252	2	9:136128000	C/G	0.84	0.93	0.92	0.02	3.4E-4	4.2E-5	5.5E-4	7.9E-8	8.0E-8	0.82	5.3E-5	2.4E-4	5.0E-6
*HBA* locus																
rs9924561	1	16:314780	G/T	0.91	0.99	–	1.4E-4	3.6E-26	5.8E-158	9.7E-87	2.4E-135	8.6E-49	4.9E-6	1.0E-11	–	–
rs76613236	2	16:230724	C/G	0.98	0.995	–	0.79	0.03	6.2E-20	2.0E-5	3.0E-18	5.3E-14	2.7E-5	1.0E-11	–	–
rs142154093	3	16:366048	C/G	0.98	0.99	–	0.02	4.0E-7	1.3E-32	2.2E-12	4.9E-33	6.5E-13	2.1E-5	1.0E-11	–	–
rs186066503	4	16:405483	T/C	–	0.994	–	0.34	8.5E-3	3.6E-30	4.4E-18	2.8E-19	2.7E-15	3.5E-6	–	1.0E-11	–
rs530159671	5	16:250184	A/G	–	0.991	0.997	4.5E-3	7.5E-8	1.7E-24	7.2E-12	2.0E-16	2.3E-7	2.2E-4	–	1.0E-11	–
rs8058016	6	16:228786	A/C	0.99	0.992	–	2.2E-3	2.8E-5	1.8E-22	1.7E-5	3.5E-21	1.0E-4	8.0E-3	1E-11	2.6E-8	–
rs60616598	7	16:297264	A/G	0.83	0.96	–	0.04	1.0E-5	8.1E-23	2.2E-11	1.8E-16	9.7E-9	2.3E-9	1.0E-11	2.8E-9	–
rs145752042	8	16:267208	A/G	0.993	–	–	1.0E-4	2.4E-7	1.2E-18	1.2E-4	3.1E-19	3.0E-4	0.04	3.2E-10	–	–
rs145546625	9	16:220583	T/C	0.98	0.93	–	0.19	0.08	2.5E-14	0.17	8.8E-15	1.4E-5	5.9E-3	0.22	1.0E-11	–
rs115415087	10	16:205132	T/C	0.018	0.004	–	0.80	0.24	1.2E-12	1.2E-4	3.5E-14	3.2E-10	0.17	1.4E-10	1.3E-3	–
rs61743947	11	16:240000	T/C	0.995	–	–	0.04	7.0E-5	2.4E-13	1.4E-11	8.7E-12	0.13	2.3E-6	3.1E-8	–	–
rs60125383	12	16:176446	A/T	0.43	0.62	0.55	0.11	0.001	3.1E-13	2.1E-6	4.5E-8	1.3E-4	0.91	6.1E-7	0.01	2.0E-5
rs55932218	13	16:221151	T/C	0.05	0.01	–	0.52	0.03	6.3E-10	1.2E-4	2.0E-8	2.1E-3	3.2E-5	5.0E-8	0.01	–
rs8051004	14	16:198835	T/C	0.10	0.05	0.02	0.81	0.04	7.2E-11	3.2E-8	8.1E-8	0.06	0.04	0.01	5.1E-7	0.04
*PLIN4/PLIN5* locus																
rs919797	1	19:4498157	A/G	0.68	0.49	0.46	5.1E-2	5.5E-4	1.9E-11	1.8E-5	6.5E-9	4.4E-1	8.8E-1	0.35	9.5E-3	1.1E-8
rs12459922	2	19:4455862	A/G	0.12	0.25	0.26	0.003	0.003	4.3E-8	0.34	1.4E-9	0.09	0.11	0.17	4.9E-5	1.0E-4

Bold font for combined-phenotype analysis indicates that the index SNP also had the lowest reported p-value for that particular trait. Variants not meeting effective heterozygosity criterion of 35 excluded. *AA* African American, *HL* Hispanic/Latino, *EU* European American. ^a^Restricted to populations with > 1000 participants

Trait-specific directions of effect were largely consistent with pairwise correlations. Among 58 independent association signals identified via conditional analysis, 64% (*n* = 37) exceeded genome-wide significance for the combined-phenotype lead SNP in two or more traits. When comparing genome-wide significant associations for two traits exhibiting a pairwise correlation >|0.2| among these loci, in 93% of instances (119 of 128) the direction of effect matched the direction of trait correlation (Fig. 1a, b, Tables S5A, S6). Eight of nine trait-pair associations with directions of effect opposite of expectation were instances in which MCH or MCV drove the lead SNP association, and HCT or HGB had a different lead SNP in high LD with the combined-phenotype lead SNP (r^2^ > 0.8 in the combined MEGA-genotyped study population). Only one of nine associations was in a trait pair exhibiting moderate correlation: HGB and RBCC (*ρ* = 0.68) exhibiting opposite directions of effect for rs9924561, the lead SNP in the *HBA1/2* region on chromosome 16.

### Evidence of independent associations at established loci

We identified 20 independent association signals at seven loci (*HFE*, *CCND3*, *HBS1L/MYB*, *CITED2*, *ABO*, *HBA1/2*, and *PLIN4/5*, Table 1, Fig. 1a). The majority of lead SNPs were common to all ancestries (MAF > 0.01); evidence of association was most significant in European Americans at *HFE* and *HBS1L/MYB* loci, whereas Hispanics/Latinos had the most significant association at both *CITED2* lead SNPs. In two instances, known causal variants accounted for the entire association signal after conditioning. At the *HFE* locus, both rs1800562 (HFE p.C282Y) and rs1799945 (HFE p.H63D, r^2^~0.99 with lead SNP rs2032451) are known coding hemochromatosis variants and accounted for all significant associations within +/− 3 Mb of the lead SNP [48]. Similarly, rs2519093 and rs10901252 are in moderate to high LD with variants that affect RBC traits but also determine an individual’s ABO blood type, and adjusting for these two variants accounted for the entire association at this locus.

**Fig. 2:**
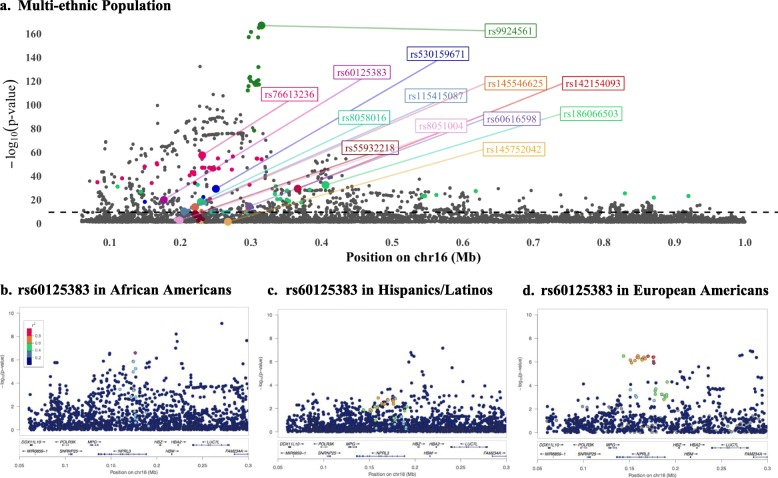
Multiple independent associations with MCH demonstrate complex genetic architecture at *HBA1*/*HBA2* locus. All plots: each point represents one SNP; x-axis: increasing position on chromosome 16 left to right; y-axis: -log_10_(*p*-value) of the association with MCH. **a** Regional association plot of 14 independent associations in unadjusted analysis of multi-ethnic study population (*n* = 41,317). Large circles represent conditionally independent lead SNPs, labeled by rsid (order of conditioning is shown in Table 1); small colored SNPs represent variants in high LD (r^2^ > 0.8 in LD in pooled MEGA subpopulation) with the lead SNP of the corresponding color. **b-d** Locus-Zoom regional association plots of MCH association with rs60125383 (11th round of conditioning, purple diamond) in African Americans on an African American LD background (**b** *n* = 8703**)**, Hispanics/Latinos on a Hispanic/Latino LD background **(c**, *n* = 17,380**)**, and European Americans on a European LD background (**d** *n* = 14,707**)**. SNP correlation with the lead SNP (r^2^) is colored according to the legend in (**b**). Annotated Refseq genes proximal to the lead SNP are shown by position above the X axis

Of note, the *HBA1/2* locus demonstrated ancestry specificity (i.e., the lead SNP was monomorphic in one or more ancestries) at 11 of 14 conditionally independent SNPs (Fig. 2a, Tables S5B-D). With the exception of rs60125383 (frequency of the A allele: 0.43 in African Americans, 0.55 in European Americans, 0.62 in Hispanics/Latinos), located in a nonsense-mediated-decay transcript for *NPRL3*, no lead SNP at this locus was common to all ancestries. The LD block for rs60125383 contained fewer variants in African Americans (Fig. 2b, no SNPs r^2^ > 0.4) compared to Hispanics/Latinos (Fig. 2c, 10 SNPs r^2^ > 0.6) and European Americans (Fig. 2d, 13 SNPs r^2^ > 0.6).

### Sensitivity analyses

Trait-specific sensitivity analyses identified two previously-unreported variants exceeded genome-wide significance for a single RBC trait in the univariate analyses, yet did not meet genome-wide significance in the combined phenotype. Rs6573766 was specific to RBCC (*p* = 1.1E-9) and is common to all ancestries but was poorly captured by earlier genotyping arrays and is not represented in 1000 genomes phase 3 data (Figure S3, Table S7). Rs145548796 was significant for MCV (*p* = 4.6E-9) and is rare (< 1%) in all populations, only meeting the inclusion criteria in the MEGA pooled sample and one study sub-population (Figure S4, Table S7). Ancestry-specific sensitivity analyses did not uncover any significant association signals that did not achieve genome-wide significance in the overall study population.

When adjusting for esv3637548 deletion dosage in the MEGA-genotyped subgroup, we observed evidence of both attenuation and strengthening of effect at otherwise conditionally independent lead SNPs at the *HBA1/2* locus (Table S8). Specifically, eight lead SNPs lost more than two orders of magnitude *p*-value after conditioning on esv3637548; one increased in significance; and five remained unchanged. Among the lead SNPs in this chromosomal region which remained significant was rs145546625, which was previously reported as significant for MCV independent of esv3637548 in a GWAS of HCHS/SOL participants using a different genotyping array [28]. All other PAGE lead SNPs in the *HBA1/2* region either did not pass QC or imputation criteria for the custom array used in that study, or had *p* > 1E-07 in the primary analysis.

### Generalization of previously reported associations

Generalization of previously identified association signals varied for trait-specific loci (*p* < 1.07E-4, Tables S9-S11), ranging from 50 of 143 (35%) for MCHC to 93 of 121 (77%) for HGB. Ancestry-specific generalization varied by trait, with the highest proportion of generalization occurring in the European-ancestry sub-population and the lowest occurring in African Americans, which may be due to power differences to detect associations by ancestry.

### eQTL function of index SNPs

To assess the potential regulatory roles of lead SNPs, we evaluated cis-eQTL (< 500 kb) associations for all lead SNPs in GTEx as available [49]. Thirty-three of 51 SNPs were low-frequency or common (MAF > 1%) in the European-ancestry GTEx population and had available information in whole blood, liver, spleen, and/or thyroid tissues. Fourteen SNPs exhibited significant associations in RBC-relevant tissues; seven SNPs were eQTLs for multiple genes (Table S12). Although approximately 40 genes were within 500 kb of each of the chromosome 16 lead SNPs, none of the lead SNPs in this region exceeded a MAF > 1% in the GTEx study population and hence could not be evaluated for cis-eQTLs.

## Discussion

RBC traits are complex quantitative phenotypes that have been broadly examined in GWAS of European- and East Asian-ancestry study populations. Here, we examine the benefits of identifying and characterizing RBC trait associations in the ancestrally diverse PAGE study population using a combined-phenotype approach. Although the combined-phenotype method we employed did not enable identification of novel loci, ancestral diversity improved characterization of loci containing both ancestry-specific and common variants. The continued underrepresentation of diverse populations in GWAS despite the growing clinical and public health significance of GWAS-enabled tools that are ancestry-specific underscores the continued importance of expanding existing RBC trait GWAS of predominantly European and East Asian populations to global populations [50–53].

With regard to regions exhibiting multiple independent significant associations, our results demonstrate allelic heterogeneity at known RBC trait loci, the characterization of which was enabled by an inclusive study design. Of particular note was our identification of eleven variants specific to African and/or Amerindian ancestries within the first megabase of chromosome 16. The chromosome 16 region includes hemoglobin genes *HBA1*, *HBA2*, *HBM*, and *HBZ* as well as fifty other protein-coding genes that should be examined for plausible roles in RBC trait biology. Decades of research have demonstrated selective pressure in this region occurring over millennia in malaria-endemic regions of the world but, as with many other complex quantitative traits, red blood cell traits—specifically with regard to the *HBA1/2* locus—have been primarily analyzed in Eurocentric study populations. Given the high polygenicity and complexity of quantitative RBC traits, our identification of over a dozen independent association signals suggests a highly-transcribed region with either complementary or redundant regulatory mechanisms that may affect multiple genes. Future work could extend our efforts by examining other populations in malaria-endemic regions, as well as previously identified and highly influential structural variants, including a previously identified 3.7 kb copy number variant, which we were only able to evaluate as a sensitivity analysis [28].

A combined-phenotype method was selected due to its purported ability to increase statistical power to identify novel loci with modest effects across multiple correlated traits. However, sample sizes of previous RBC trait GWAS suggest that many loci with modest effects and lead SNPs in the low to common allele frequency range in European or East Asian populations have already been identified. Power was also lacking to detect loci that might be specific to other race/ethnic groups—although African Americans and Hispanics/Latinos were well-represented in this study, sample sizes similar to European populations will not be proportionately representative of genetic diversity, particularly for variants that are low-frequency or difficult to impute. This observation demands an increase in representation of African Americans and Hispanics/Latinos, as narrower (on average) LD blocks in populations exhibiting ancestral admixture also improve fine-mapping for prioritizing candidate variants for functional characterization. A combined-phenotype method can also improve the interpretability of association signals when one causal SNP per association signal is assumed. For example, a direction of effect inconsistent with the phenotypic correlation of two RBC traits is feasible in some anemia states, for which MCV and RDW—despite being negatively correlated in healthy individuals—may vary widely depending on the underlying cause [54, 55]. The African-ancestry-specific SNP rs9924561 (previously identified for MCH, MCHC, and MCV) is an example of a variant that unexpectedly showed opposite directions of effect for HGB and RBCC (pairwise correlation = 0.68) in our study [28, 30, 56]. The mechanism driving very strong associations (*p* < 1E-15 in all traits aside from HCT) with this intronic variant remains uncharacterized, likely because it is not present in European-ancestry populations and hence could not be detected in otherwise highly powered studies [12, 31]. The identification of such candidate functional variants for multiple traits with the added context of the phenotypic correlation can provide insight for molecular experimentation examining causal biological mechanisms.

The possibility that combined-phenotype methods could benefit the study of other correlated polygenic traits still merits further investigation, particularly with groups of traits that may overlap in genetic architecture, but have not been previously examined in concert. Over the past three decades, RBC traits have been associated with cardiovascular disease outcomes like heart failure and stroke, highlighting the potential for identifying novel pleiotropic loci [6, 57–62]. Indeed, combined-phenotype approaches that examine the shared genetic architecture underlying intermediate phenotypes and clinical events may be particularly powerful for outcomes like stroke and heart failure, given that phenotypic heterogeneity of these phenotypes has complicated locus identification and characterization.

Our evaluation of lead SNPs’ effects on expression in RBC-relevant tissues faced known constraints that limited interpretation and contextualization of identified variants. Crucially, the vast majority of publicly available functional data were collected from European-ancestry individuals, precluding the use of these databases for interpreting potential effects of ancestry-specific or low-frequency SNPs on gene expression. For example, rs8051004 is one of two less frequent variants that were detected in European-ancestry populations at the *HBA1/2* locus (CAF = 0.02). However, rs8051004 was reported as “monoallelic” in spleen tissue in GTEx, despite having a 10% allele frequency in PAGE African Americans and 12 and 11% in the 1000G African and East Asian superpopulations, respectively. The exclusion of populations with African, Amerindian, and Asian ancestry continues to hamper the potential benefits of these resources. Additionally, while the GTEx consortium has made extensive efforts to characterize a wide array of tissue types, bone marrow was not included [49]. RBCs enucleate in the bone marrow prior to entering circulation, with no nuclear transcription and extremely limited translation occurring in mature RBCs. Therefore, bone marrow is the only tissue for which eQTL data characterizing the effects of genetic variation on gene expression for RBCs directly.

As with other genetic association studies, we faced several limitations. First, sample sizes for RBC trait GWAS have ballooned to nearly 200,000 participants and we were restricted to a smaller study population. However, the PAGE study has recently demonstrated that modest-sized studies that are more ancestrally diverse improve detection of novel and independent signals compared to simply increasing the number of European-ancestry individuals [56]. Second, while this study did improve on previous studies in terms of representation from African and American continental ancestries, we were unable to evaluate associations in several populations, particularly South Asians, Pacific Islanders, Native Americans, and Native Hawaiians. Native Americans and Native Hawaiians are represented in PAGE, but RBC phenotypes were not measured in contributing studies. South Asian study populations have been included in several previous RBC trait GWAS; Native Americans and Pacific Islanders remain underrepresented in GWAS of all complex traits [15, 20, 39, 63]. Third, we were unable to evaluate structural variants, which have traditionally been difficult to impute, and re-calling all structural variants within significant loci was outside the scope of this work. A sensitivity analysis accounting for the effect of esv3637548 in MEGA-genotyped study participants suggests that further evaluation is required to determine whether true causal variants overlap the position of this 3.7 kb structural variant on other ancestral haplotypes. However, it is expected that some structural variants will be adequately represented by proxy SNPs, and future sequencing-based studies will be able to characterize these rare variants. Finally, eQTL data could not be comprehensively interpreted given the limitations of publicly available databases as described above. It is imperative that these resources focus their efforts on improving inclusivity over the next several years to keep abreast of increased representativeness in association studies.

## Conclusion

In conclusion, we identified over 50 association signals within 39 loci in a combined-phenotype analysis of seven RBC traits. We did not observe large improvement in discovery signal detection by using the combined-phenotype methods, although further work is required to fully test the utility of these approaches. However, our work demonstrates the benefits of diverse study populations for highly polygenic traits, in spite of the fact that while global populations are increasing in genetic diversity, genetic research has become less diverse. As genomics tools become more broadly available, our results underscore the critical importance of including diverse global populations so the benefits of genomics research can be equitably applied.

## Methods

### Study population

The PAGE study comprises ancestrally-diverse study populations from United States cohorts and biobanks evaluating common complex diseases and accompanying risk factors (see online supplement for more information). This study used data from self-reported African American, Asian American, European American, Hispanic/Latino, and Native American participants from the Atherosclerosis Risk in Communities Study (ARIC); the Coronary Artery Risk Development in Young Adults Study (CARDIA); the Hispanic Community Health Study/Study of Latinos (HCHC/SOL); the Icahn Mt. Sinai School of Medicine BioME Biobank (BioME); and the Women’s Health Initiative (WHI, described above). Our study population comprised sixteen analytic subgroups which were genotyped and imputed separately. Fifteen of the sixteen analytic subgroups were identified by study and self-reported race/ethnicity (Tables S2, S3). The sixteenth subgroup was a pooled sample of self-reported African American, Asian American, Hispanic/Latino, Native American, and “Other” MEGA-genotyped individuals from BioMe, HCHS/SOL, and WHI. Participants were excluded if they had ever been diagnosed with HIV or leukemia, were pregnant at time of blood draw, were receiving chemotherapy at time of blood draw, or had a severe hereditary anemia (primarily sickle-cell disease, determined by genotype).

### RBC trait measurement

RBC traits were measured with hemanalyzers following standardized laboratory protocols from blood draws at the earliest available visit (see online supplement) for the three primary (HCT, HGB, and RBCC) and four derived (MCH, MCHC, MCV, and RDW) RBC traits (Table S1). RBC trait values that exceeded four standard deviations from the mean of the trait in the overall study population were excluded, mirroring protocols established by prior GWAS [28, 45]. Pairwise correlation coefficients were calculated in the MEGA-genotyped analytic subgroup (see below) adjusting for all the covariates used in univariate regression analysis, specifically age at blood draw, sex, study site or region, and ancestral principal components.

### Genotyping, quality control, and imputation

Genotyping methods have been described for each of our study sub-populations previously; all imputation of genotype data used in this study was performed by the PAGE coordinating center [64]. Briefly, genotyping arrays and quality control measures used were as follows. Affymetrix Genome-Wide Human SNP Array 6.0 for ARIC, BioMe Mt. Sinai Biobank European Americans, CARDIA, and WHI SHARe. The Illumina OmniExpress was used to genotype individuals for all remaining BioMe Mt. Sinai Biobank participants. WHI GARNET participants were genotyped on the Illumina Human Omni1-Quad v1–0 B array; WHI GECCO participants on the Illumina 610 K and Cytochip 370 K arrays; WHI HIPFX participants on the Illumina 550 K and 610 K arrays; WHI LLS participants on the Illumina HumanOmniExpressExome-8v1_A array; WHI MOPMAP participants on the Affymetrix Gene Titan, Axiom Genome-Wide Human CEU I Array Plate; and WHI WHIMS participants on the HumanOmniExpress Exome-8v1_B array. All remaining participants from BioMe, HCHS/SOL, and WHI were genotyped on the Illumina Infinium Expanded Multi-Ethnic Genotyping Array (MEGA).

With regard to quality control, studies employed either a 90% (ARIC, MOPMAP) or 98% (all other studies) SNP call-rate threshold. A sample call rate of 95% was employed for ARIC and. A 98% rate for MEGA-genotyped participants, with no sample call rate applied to remaining studies. Similarly, a 1E-06 HWE *p*-value threshold was employed for ARIC, and a 1E-04 threshold for MEGA-genotyped participants. Additional study-specific genotype QC criteria are described in Table S2. All studies were imputed to the 1000 Genomes phase 3 reference panel by the PAGE coordinating center after study-specific quality control criteria were applied (Table S2, 56). We further excluded SNPs on a sub-study-specific basis which had poor imputation quality (< 0.4) or an effective heterozygosity < 35 (calculated as 2 x CAF x (1-CAF) x N x imputation quality, where CAF is coded allele frequency and N is sample size).

## Statistical methods

### Overall reporting of results

Previously-reported SNPs for the seven RBC traits evaluated in this study were identified through review of the NHGRI-EB GWAS Catalog [65] as of January 1, 2019, supplemented by a PubMed search. Multi-ethnic combined-phenotype results were presented as the primary findings, employing Bonferroni correction assuming 10 M independent tests (i.e., genome-wide significance refers to p_aSPU_ < 5E-9). We defined a locus using physical proximity (+/− 500 kb from the lead SNP), and we defined an association signal as the lead (most significant) SNP and proxy SNPs in local LD based on conditional independence within ten megabases. Discovery loci were defined as ≥500 kb from and conditionally independent of a variant previously reported to satisfy the field standard *p* < 5E-8 for any of the seven RBC traits. Ancestry-specific and trait-specific analyses were performed as sensitivity analyses to improve interpretation of results. Complete summary-level results are available through dbGaP (phs000356).

### Univariate analysis

Univariate associations for the seven RBC traits were estimated assuming an additive genetic model of inheritance and adjusting for linear effects of age at blood draw, sex, study site or region, and ancestral principal components [66]. The total MEGA-genotyped subgroup was analyzed using generalized estimating equations allowing correlated errors for first or second-degree relatives, and independent error distributions by self-reported ancestry group [67]. Linear regression was implemented in SUGEN for the other 15 analytic subgroups [67]. For each RBC trait, METAL software was used to perform inverse-variance-weighted meta-analysis across all sub-studies [68]. SNP effect heterogeneity was measured with the Cochran’s Q test. SNP meta-analysis *p*-values were assessed by RBC trait by calculating genomic inflation factors (λ) and plotting the expected distribution against observed results.

### Combined-phenotype analyses

We used an adaptive sum of powered scores (aSPU) simulation-based method to perform a combined-phenotype analysis incorporating univariate results from seven RBC traits in sixteen analytic subgroups that were combined using inverse-variance-weighted meta-analysis. To evaluate evidence for shared genetic effects across all seven RBC traits, we combined meta-analyzed univariate results with aSPU to generate a combined-phenotype *p*-value for each SNP [28, 69]. In comparison with other available methods, we chose aSPU because it exhibited low type 1 error rate in simulations; accommodated direction of effect; and was computationally scalable to the millions of SNPs measured using 1000 Genomes Phase 3 imputed data [70]. We implemented aSPU using Julia 1.0 to optimize efficiency (https://github.com/kaskarn/aspu_julia).

aSPU incorporated univariate summary z-scores, calculated for each SNP across all 7 traits, to yield a single p-value evaluating whether one or more of the traits were associated with a given SNP. Briefly, the procedure estimates Σ, the 7 × 7 correlation of null z-scores across univariate results and draws 10^11^ Monte-Carlo samples from the multivariate ${N}_7\left(0,\hat{\Sigma}\right)$ distribution. For each SNP *j*, the results for all 7 traits *z*_*j*1_, …, *z*_*j*7_ are used to form a sequence of sums of powered scores: $SPU\left(\gamma \right)={z}_1^{\gamma }+\dots +{z}_7^{\gamma }$, where γ = 0, 1, …, 8, plus *SPU*(∞) = max  ∣ *S*_7_∣. Each powered score is compared to the distribution of the 10^11^ powered scores calculated using simulated null values with the same γ to calculate a Monte-Carlo p-value. An overall SNP p-value (*p*_*aSPU*_, possible range: [1/(1 + 10^11^), 1]), is calculated by comparing the minimum p-value across the sequence of powered scores to the reference distribution of minimum *p*-values across the sequence of powered scores computed using the simulated null data. The adaptive aspect of the test lies in the potential for different γ values to yield the maximal SPU across SNPs, maintaining power compared to a test with only a single possible alternative hypothesis.

### Sensitivity analyses

Sensitivity analyses were performed for combined-trait results by self-reported race/ethnicity among analytic subgroups with greater than 1000 participants (i.e., restricted to African Americans, Hispanics/Latinos, and European Americans). Given the number of known ancestry-specific variants driving blood trait values, it was necessary to ensure that all self-reported race/ethnic groups be evaluated individually for associations that may be undetectable in the larger population. Meta-analyses of univariate summary statistics followed by combined-phenotype analysis were performed within each self-reported race/ethnicity using the same methods described above for the overall study population to identify genome-wide association signals (*p* < 5E-09).

We also examined whether there was evidence of significant trait-specific loci that were not identified in combined-phenotype analyses. Meta-analyses of each univariate RBC trait across all analytic subpopulations, as described above, were evaluated for association signals exceeding genome-wide significance (*p* < 5E-09). Although RBC traits are expected to share genetic underpinnings, particularly within pairs of correlated traits, association signals which were trait-specific in the well-powered UK BioBank blood trait GWAS suggest that each trait has its own unique suite of associations [12].

Finally, in an attempt to examine the influence of the previously identified 3.7 kb structural variant esv3637548 in the *HBA1/2* region of chromosome 16, we also adjusted for esv3637548 dosage (r^2^ = 0.86) in the MEGA-genotyped subgroup [28]. This structural variant either overlaps or has the potential to affect chromatic accessibility for multiple variants at this locus, but is present as both a duplication and a deletion. The duplication was not able to be imputed, and the deletion only met imputation quality criteria in the MEGA-genotyped study population, hence esv3637548 could not be evaluated within the entire study population in which this variant may be present. To evaluate the potential effect of this variant on each lead SNP reported as independent within our study, unadjusted combined-phenotype *p*-values were therefore compared to p-values after conditioning on esv3637548.

### Generalization of previously reported associations to PAGE

All SNPs located within 500 kb of a variant previously reported for any RBC trait were evaluated for evidence of association in the combined-phenotype analysis as well as each individual trait analysis. A generalization significance threshold of 1.07E-4 was calculated using Bonferroni correction for the previous number of one-megabase genomic regions for which one or more genome-wide-significant variants were reported for one or more RBC traits (*n* = 466, representing 1308 index SNPs previously reported for one or more of the seven RBC traits we evaluated). We first reported trait-specific associations—i.e., index variants that have been reported by trait. We did not report loci containing a SNP that exceeded genome-wide-significance for the first time in one RBC trait but were previously reported for another trait as discovery associations; therefore, we also used the aforementioned significance threshold to evaluate generalization of association signals in each trait across all known loci.

### Identification of conditionally independent association signals

Iterative conditional analysis was performed to identify all independent, genome-wide-significant combined-phenotype lead SNPs as described above. To avoid identifying SNPs as independent that were in long-range LD, we began by conditioning on the top SNP within ten megabase windows on each chromosome. To identify independent SNPs, linear models were extended to include all PAGE combined-phenotype lead SNPs on shared chromosomes using the same methods described above for univariate analysis, with an added covariate to include the dosage information for each participant at each lead SNP. Following each round of conditioning, aSPU was re-run on conditioned results. Additional rounds of conditional analyses were performed as an iterative process until no genome-wide-significant SNPs remained in the combined phenotype analysis.

## Publicly available expression quantitative trait locus (eQTL) analysis

To help prioritize candidate causal gene-variant associations at identified loci, we evaluated all available lead SNPs within significant loci in relevant available tissues (whole blood, liver, spleen, and thyroid) for evidence of association with gene expression using the Genotype Tissue Expression (GTEx) portal [49].

## Supplementary information

**Additional file 1: Figure S1.** Manhattan and Quantile-Quantile plots for individual RBC traits in the total study population. In Manhattan plots, previously reported loci (published index SNP reported *p* < 5E-08 within 500 kb of PAGE combined-phenotype lead SNP) are shown in purple; previously unreported loci with a PAGE lead SNP *p* < 5E-09 are shown in green. In Q-Q plots, all (black) *p*-values and p-values for variants > 500 kb from a previously reported significant variant *for any RBC trait* (blue) are both shown. **Figure S2.** Evidence of genetic associations shared across correlated RBC traits. X-axis: chromosome and position (top) and rsid (bottom) for each combined-phenotype lead SNP. Y-axis: trait-specific –log_10_(p-values), with increased intensity representing higher significance, for each combined-phenotype lead SNP. *P*-values scaled to a maximum –log_10_ value of 25 for improved interpretation. **Figure S3.** Locus-Zoom plots of the association between rs6573766 and RBCC in PAGE African Americans on an African American LD background (A), Hispanics/Latinos on a Hispanic/Latino LD background (B), and European Americans on a European LD background (C). Each point represents one SNP; x-axis: increasing position on chromosome 14 left to right; y-axis: -log_10_(p-value) of the association with MCH SNP correlation with the lead SNP (r^2^) is colored according to the legend in Figure S3A. Annotated Refseq genes proximal to the lead SNP are shown by position above the X axis. **Figure S4.** Locus-Zoom plot of the association between MCH (A) and MCV (B) and rs145548796 in the total MEGA study population. Each point represents one SNP; x-axis: increasing position on chromosome 6 left to right; y-axis: -log_10_(p-value) of the association with MCH SNP correlation with the lead SNP (r^2^) is colored according to the legend in Figure S4A. Annotated Refseq genes proximal to the lead SNP are shown by position above the X axis.

**Additional file 2. **Twelve supplemental tables supporting findings reported in the main text. Tables cover trait, genotyping, and QC description; ancestry- and trait-specific findings for combined-phenotype lead SNPs; sensitivity analysis of a deletion at the *HBA1/2* locus; generalization of previously reported findings to PAGE study populations; and eQTL findings for PAGE lead SNPs in relevant tissue types.

## Data Availability

Complete summary level results are available through dbGaP (http://www.ncbi.nlm.nih.gov/projects/gap/cgi-bin/study.cgi?study_id=phs000356).

## Abbreviations

ARIC: Atherosclerosis Risk in Communities study
aSPU: adaptive sum of powered scores
CARDIA: Coronary Artery Risk Development in Young Adults Study
eQTL: expression quantitative trait locus
GTEx: Genotype Tissue Expression project
GWAS: Genome-wide association study
HCHS/SOL: Hispanic Community Health Study/Study of Latinos
HCT: Hematocrit
HGB: Hemoglobin concentration
kb: kilobase
LD: Linkage disequilibrium
MCH: Mean corpuscular hemoglobin
MCHC: Mean corpuscular hemoglobin concentration
MCV: Mean corpuscular volume
MEGA: Multi-ethnic genotyping array
PAGE: Population Architecture using Genomics and Epidemiology study
RBC: Red blood cell
RBCC: Red blood cell count
RDW: Red cell distribution width
SNP: Simple nucleotide polymorphism
WHI: Women’s Health Initiative study
