# Supplementary material for: Ancestry-specific associations identified in genome-wide combined-phenotype study of red blood cell traits emphasize benefits of diversity in genomics
Source: BMC Genomics. 2020 Mar 14;21:228. doi: 10.1186/s12864-020-6626-9 (PMC7071748; doi:10.1186/s12864-020-6626-9)

**Figure S1.** Manhattan and Quantile-Quantile plots for individual RBC traits in the total study population.

In Manhattan plots, previously reported loci (published index SNP reported p<5E-08 within 500kb of PAGE combined-phenotype lead SNP) are designated in purple; previously unreported loci with a PAGE lead SNP p<5E-09 are shown in green. In Q-Q plots, all (black) p-values and p-values for variants >500kb from a previously reported significant variant *for any RBC trait* (blue) are both shown.

**A. HCT (N=67,885)**


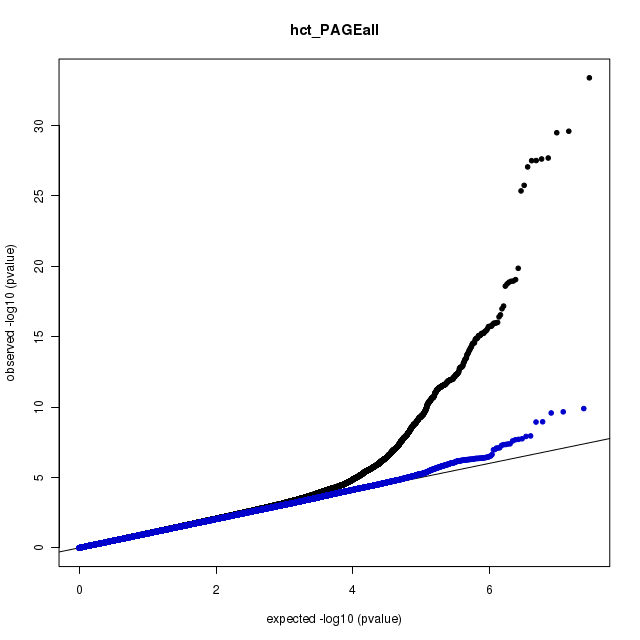

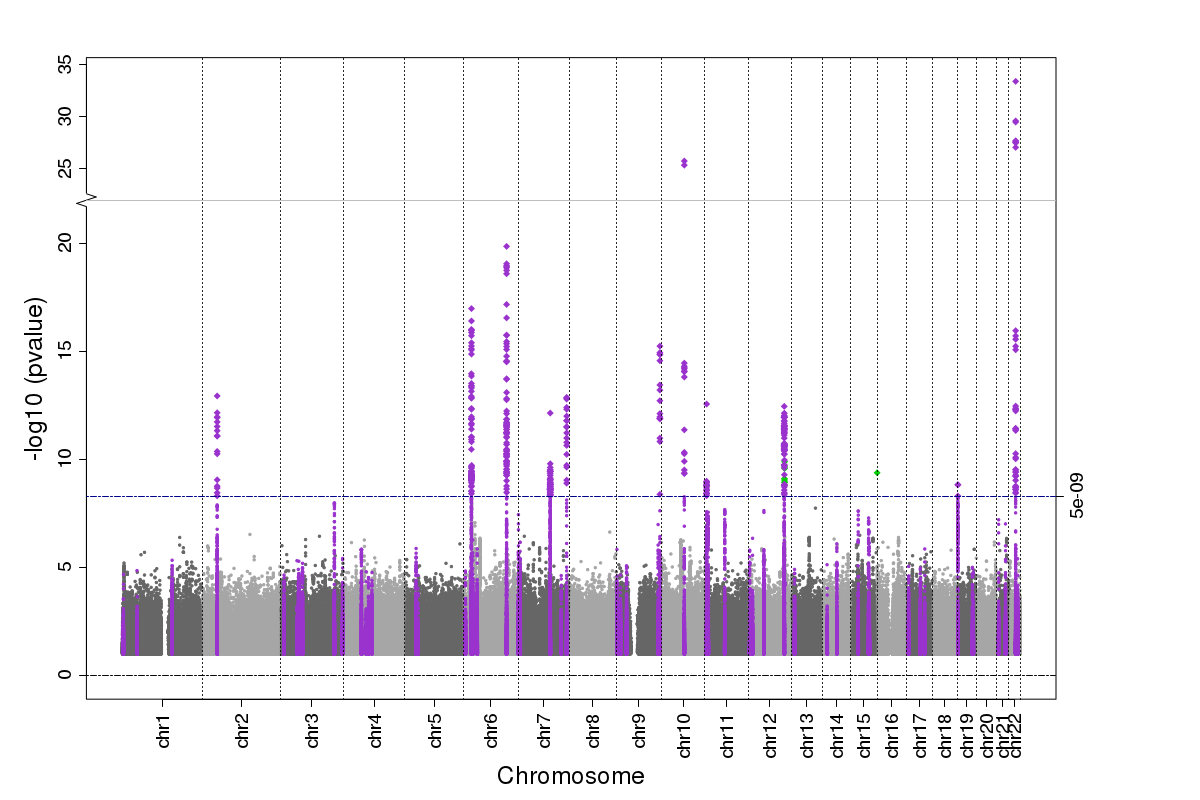


**B. HGB (N=67,870)**


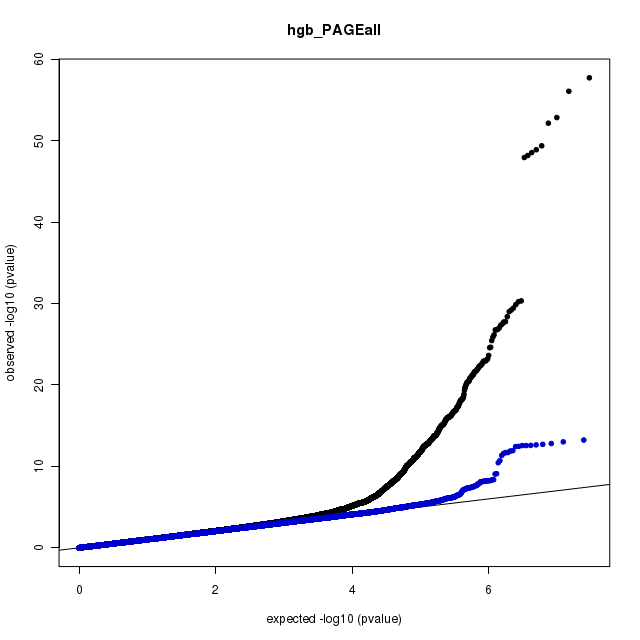

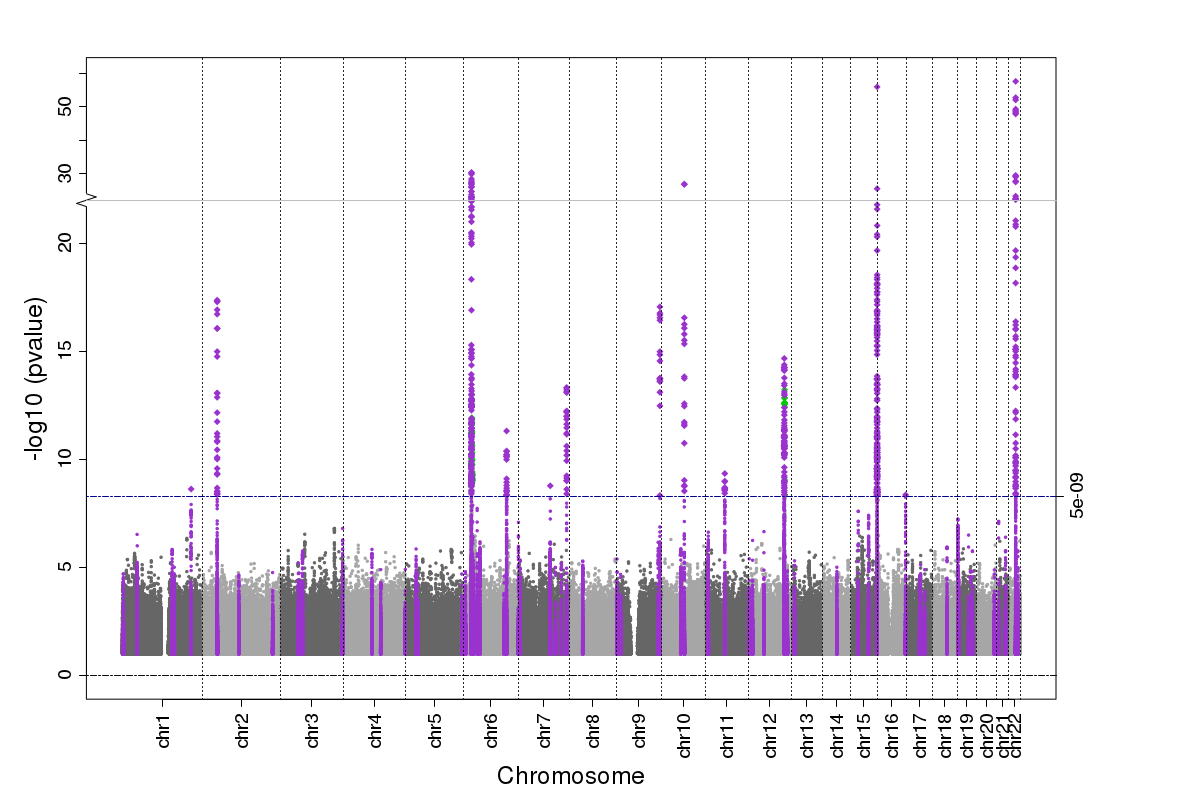


**C. MCH (N=41,317)**


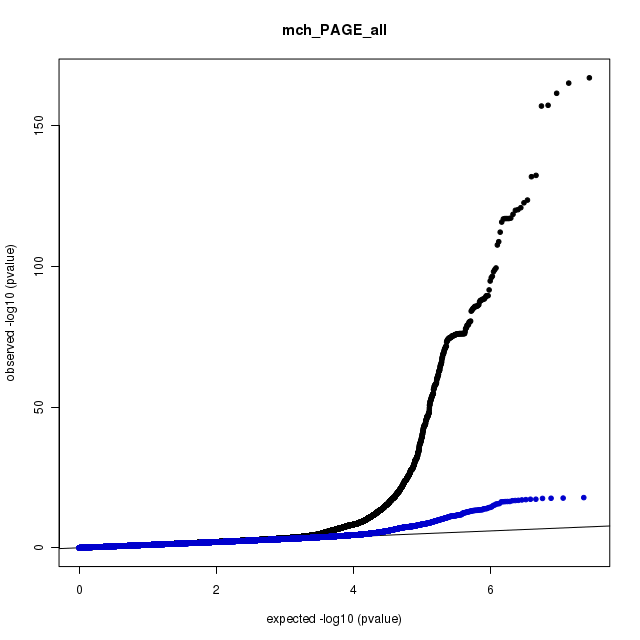

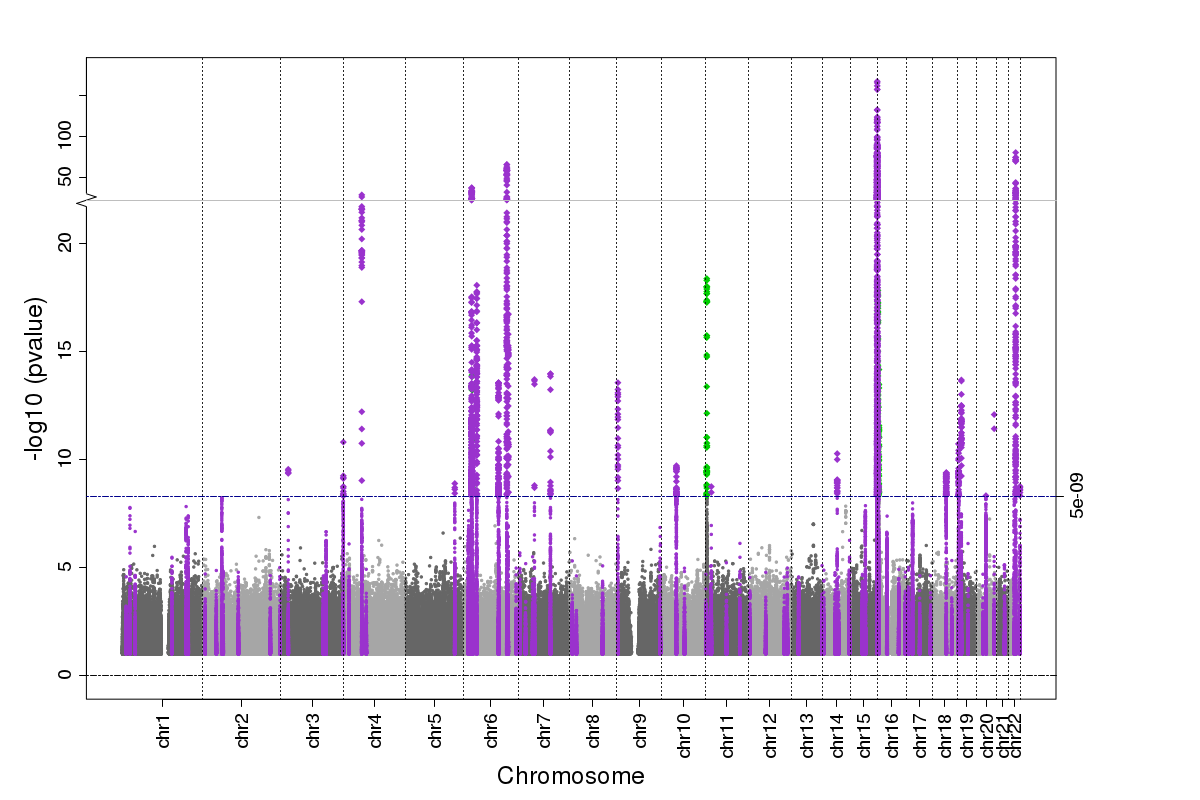


**D. MCHC (N=67,856)**


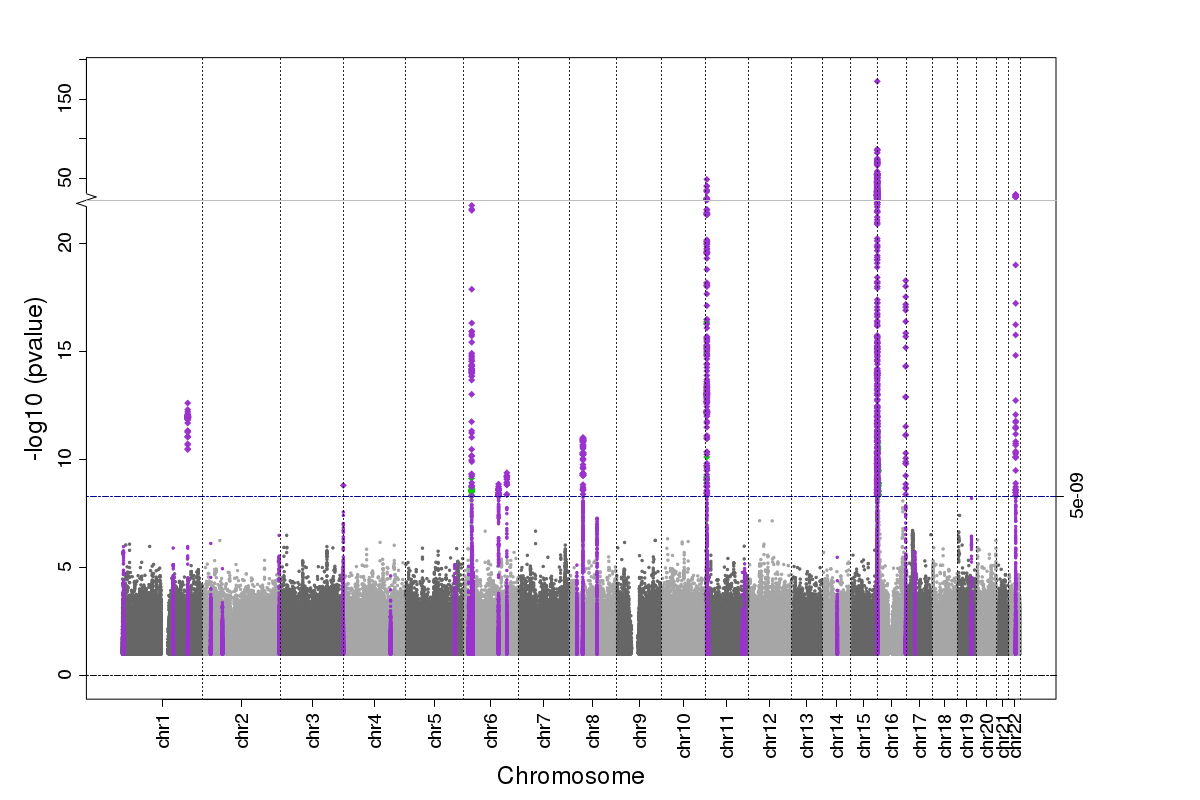

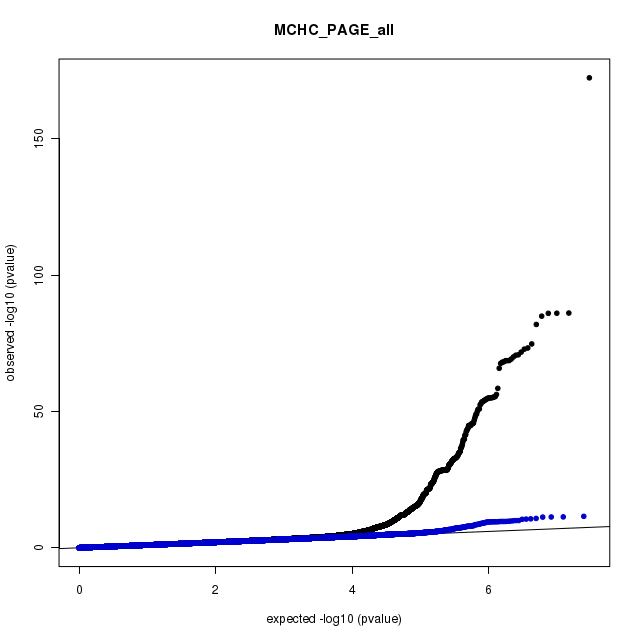


**E. MCV (N=41,276)**


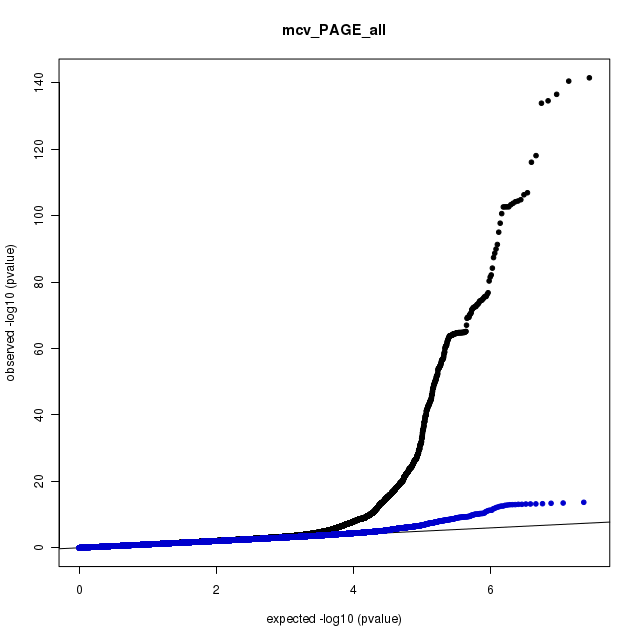

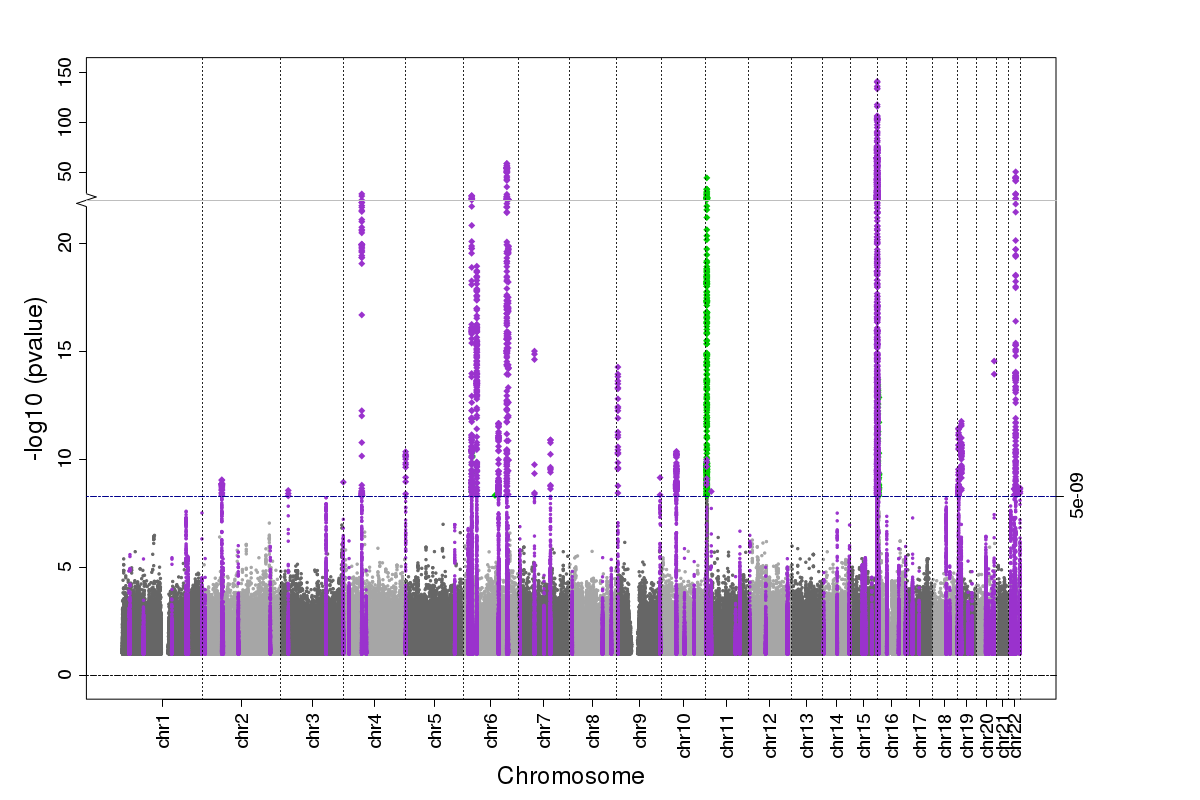


**F. RBCC (N=41,310)**


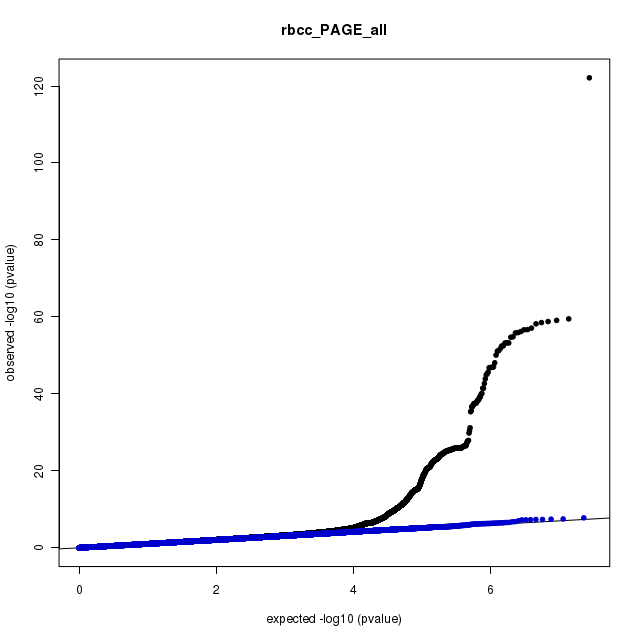

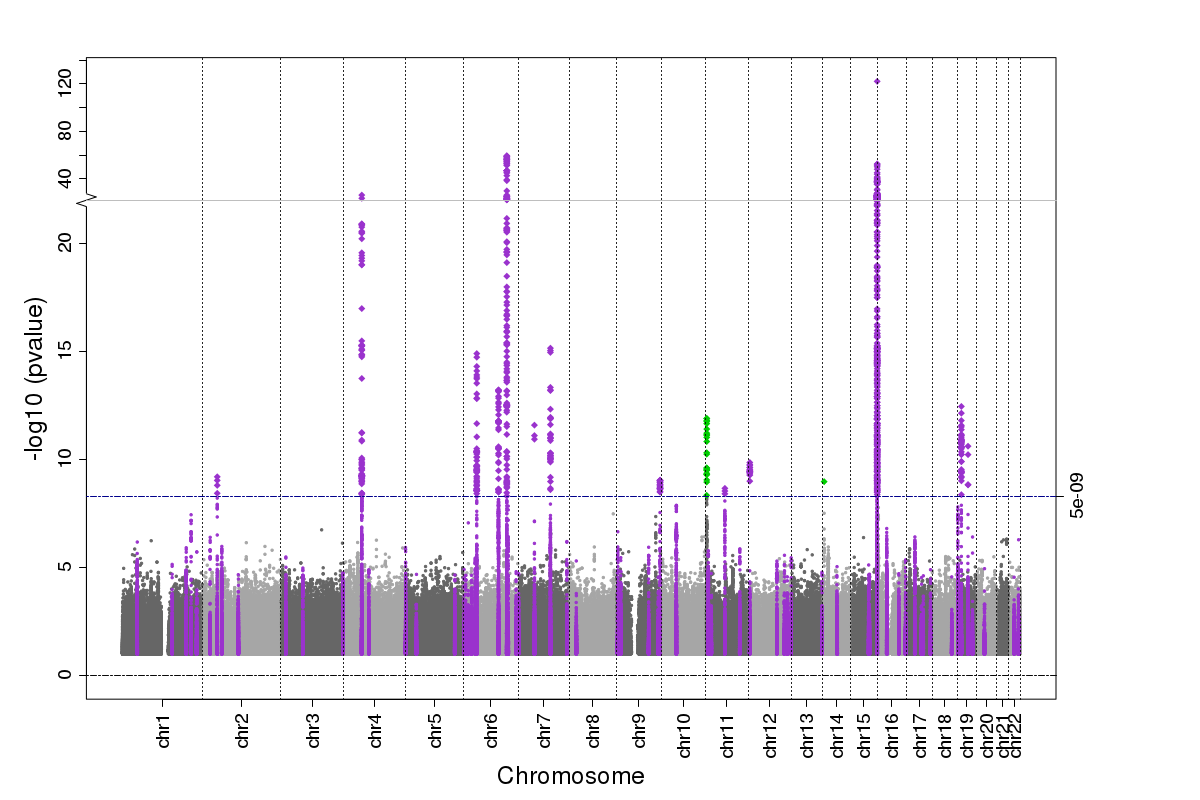


**G. RDW (N=33,549)**


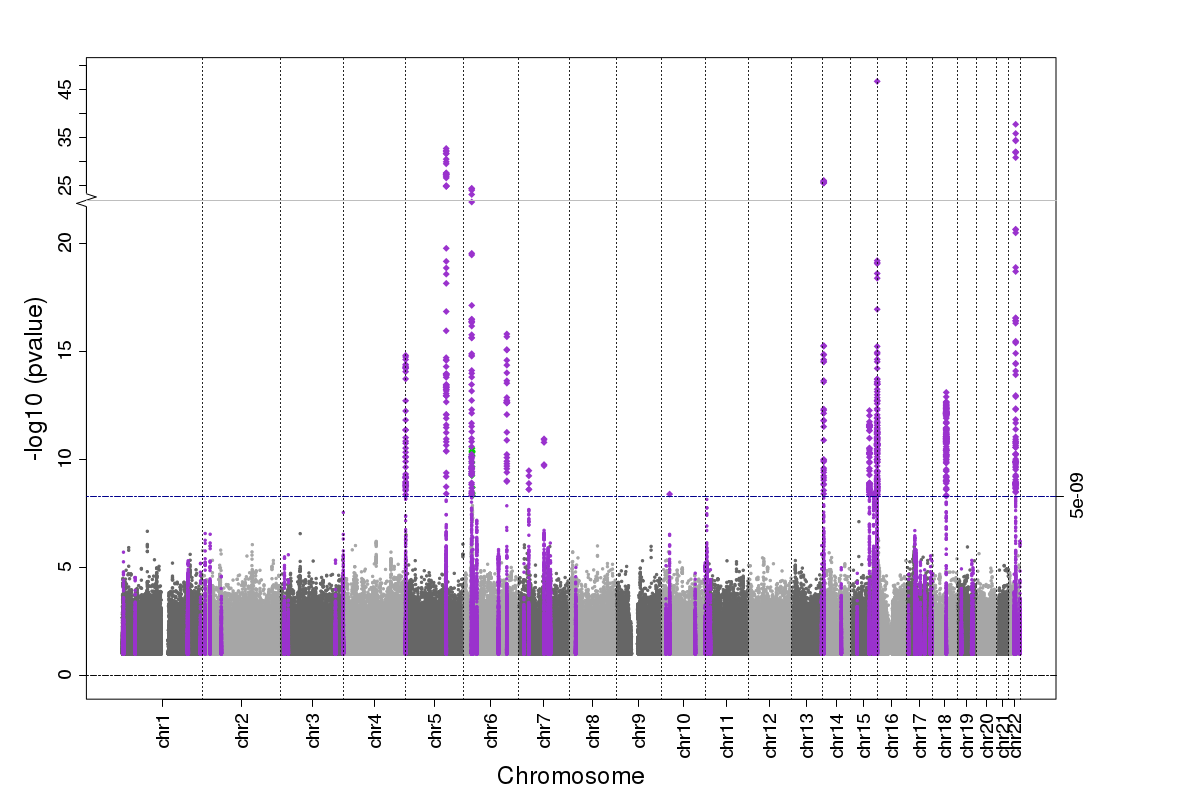

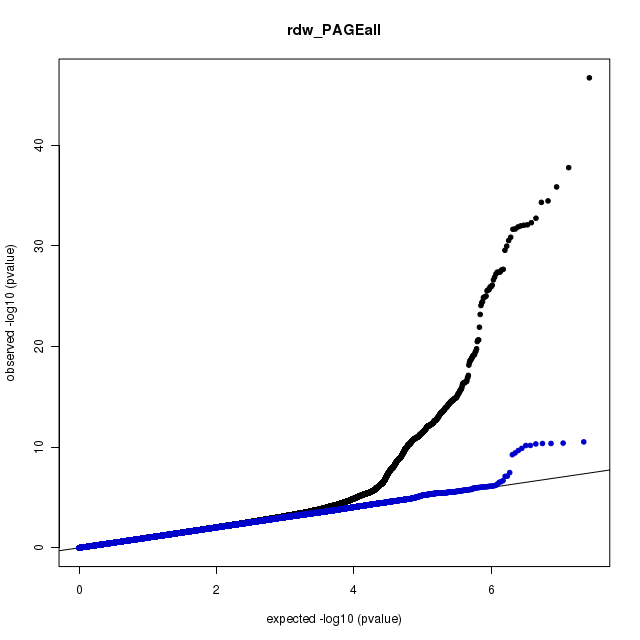


**Figure S2.** Evidence of genetic associations shared across correlated RBC traits.

X-axis: chromosome and position (top) and rsid (bottom) for each combined-phenotype lead SNP. Y-axis: trait-specific –log_10_(p-values), with increased intensity representing higher significance, for each combined-phenotype lead SNP. P-values scaled to a maximum –log_10_ value of 25 for improved interpretation.


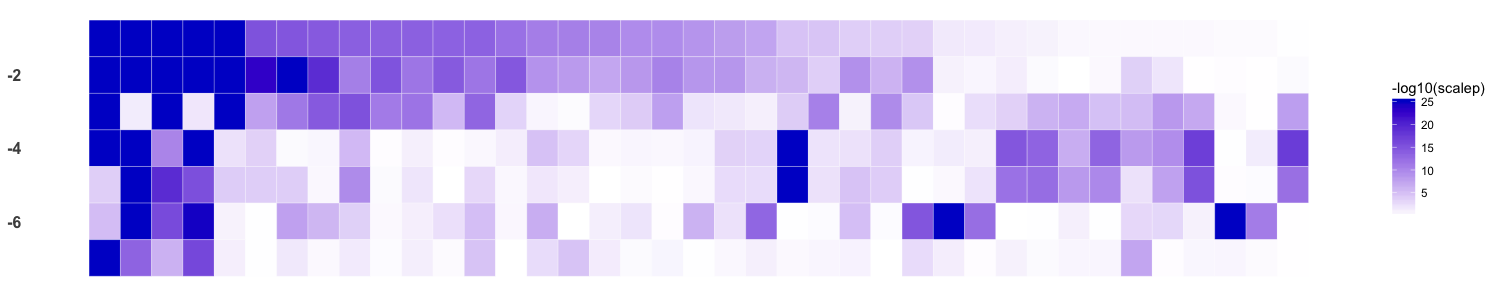

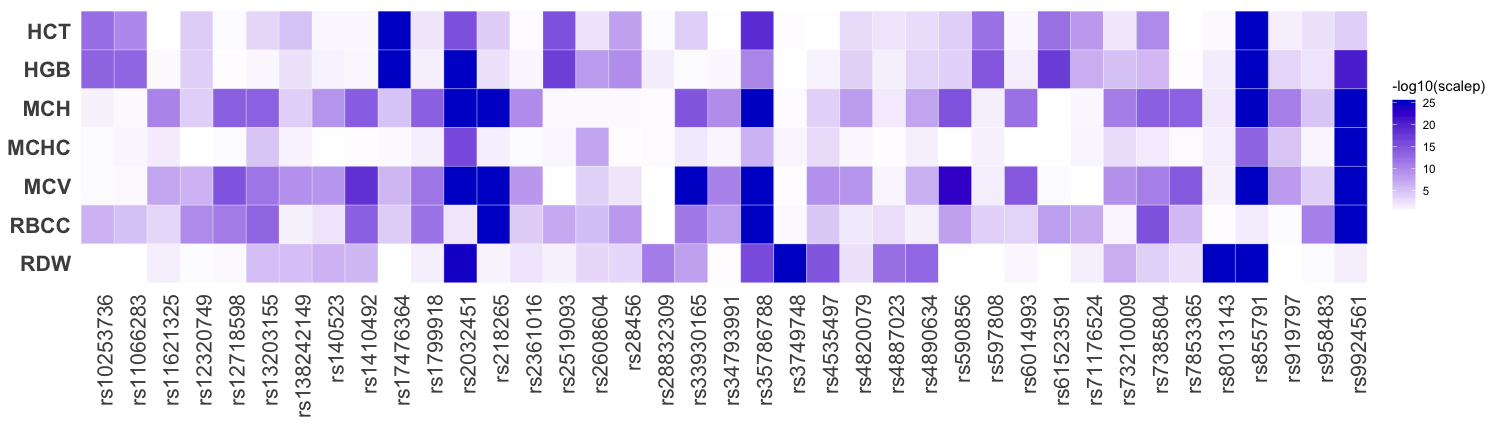


19:2177925

9:136141870

7:80319938

5:127350549

7:151415256

2:46372781

12:112840766

15:78535437

14:23494277

12:111973358

11:61589481

16:88849421

19:33759240

10:71094504

12:4331647

18:43854259

14:65472241

19:4498157

3:24341268

22:50962782

22:32879617

3:195830276

11:5860096

5:1107428

10:46080590

7:100235970

19:13002400

6:109614844

9:4855858

20:55991637

7:50428445

6:41907855

6:139844429

4:55408999

6:26092170

11:5248233

22:37462936

6:135419042

16:314780


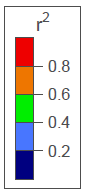


**Figure S3.** Locus-Zoom plots of the association between rs6573766 and RBCC in PAGE African Americans (A), Hispanics/Latinos (B), and European Americans (C)

**C.** RBCC-rs6573766 association in PAGE European American study participants

**A.** RBCC-rs6573766 association in PAGE African American study participants

**B.** RBCC-rs6573766 association in PAGE Hispanic/Latino study participants


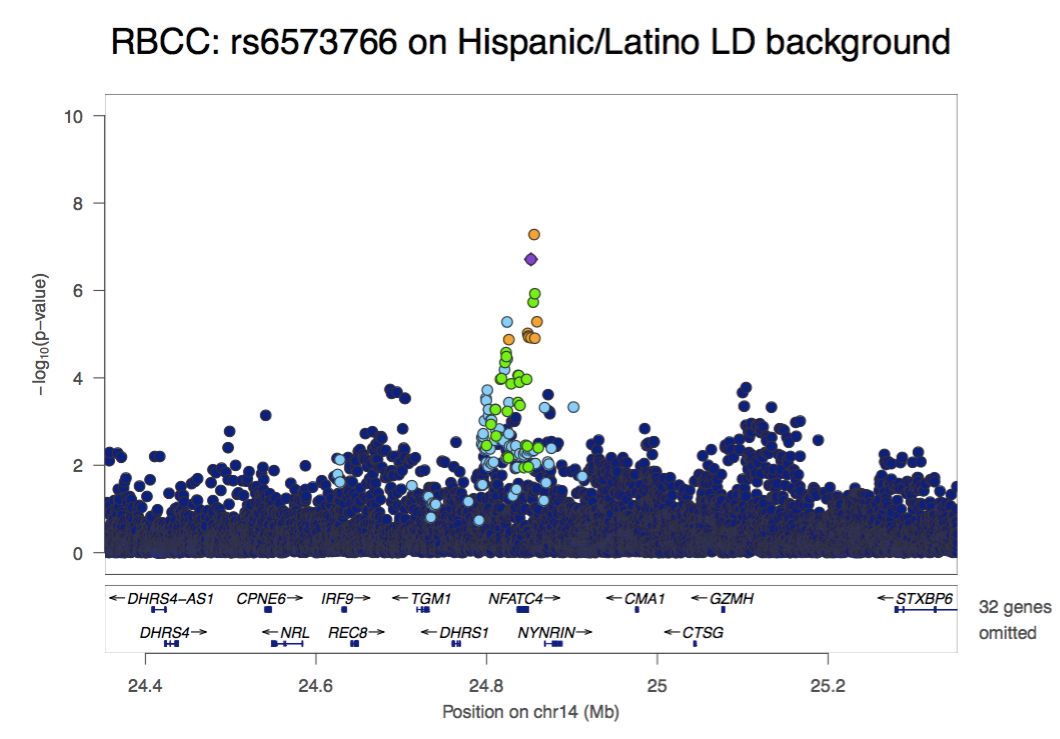

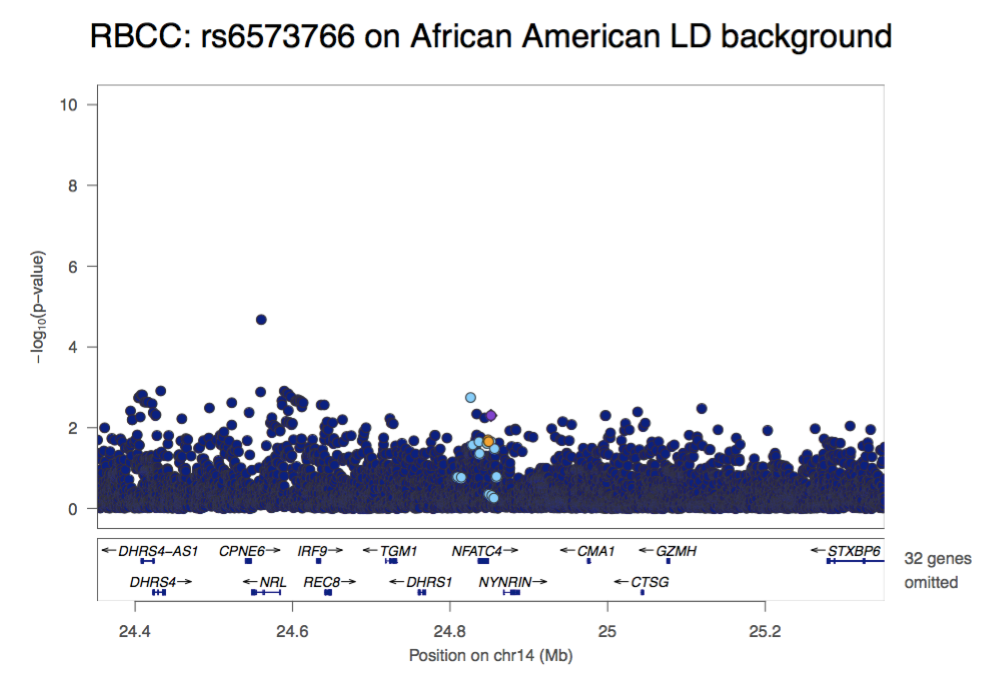

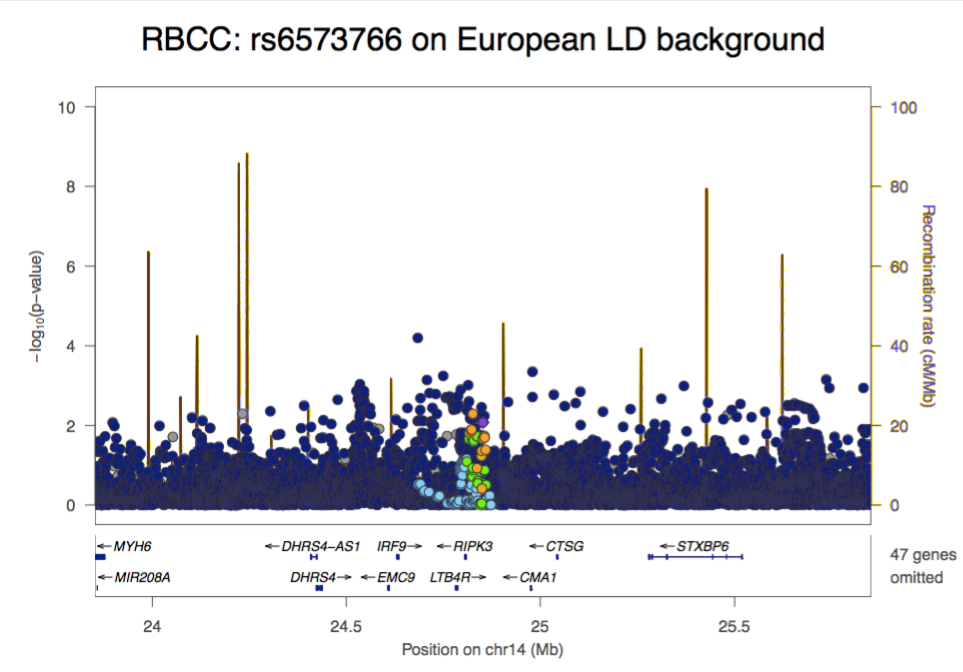


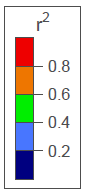


**Figure S4.** Locus-Zoom plot of the association between MCH (A) and MCV (B) and rs145548796 in the total MEGA study population

**A.** MCH-rs145548796 association in MEGA-genotyped study participants


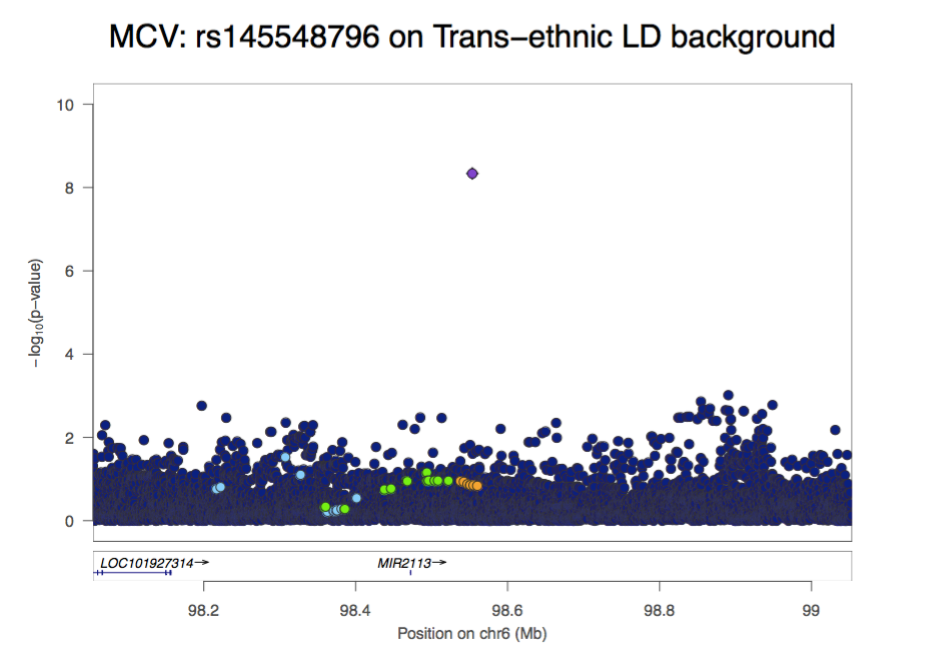


**B.** MCV-rs145548796 association in MEGA-genotyped study participants


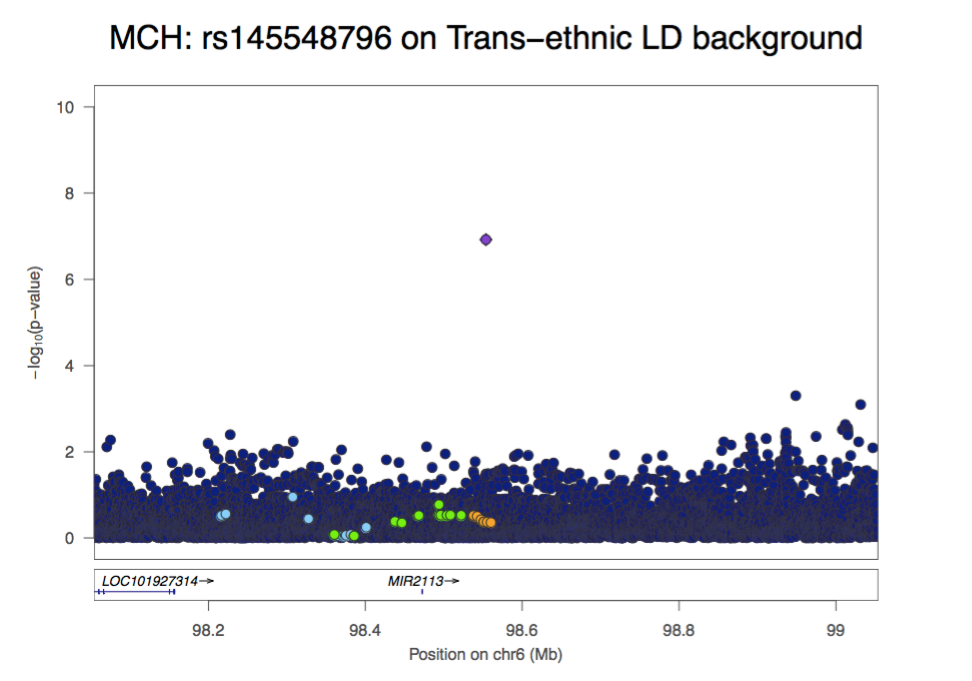

Supplement: Supplementary file 1 — Additional file 1: Figure S1. Manhattan and Quantile-Quantile plots for individual RBC traits in the total study population. In Manhattan plots, previously reported loci (published index SNP reported p < 5E-08 within 500 kb of PAGE combined-phenotype lead SNP) are shown in purple; previously unreported loci with a PAGE lead SNP p < 5E-09 are shown in green. In Q-Q plots, all (black) p-values and p-values for variants > 500 kb from a previously reported significant variant for any RBC trait (blue) are both shown. Figure S2. Evidence of genetic associations shared across correlated RBC traits. X-axis: chromosome and position (top) and rsid (bottom) for each combined-phenotype lead SNP. Y-axis: trait-specific –log10(p-values), with increased intensity representing higher significance, for each combined-phenotype lead SNP. P-values scaled to a maximum –log10 value of 25 for improved interpretation. Figure S3. Locus-Zoom plots of the association between rs6573766 and RBCC in PAGE African Americans on an African American LD background (A), Hispanics/Latinos on a Hispanic/Latino LD background (B), and European Americans on a European LD background (C). Each point represents one SNP; x-axis: increasing position on chromosome 14 left to right; y-axis: -log10(p-value) of the association with MCH SNP correlation with the lead SNP (r2) is colored according to the legend in Figure S3A. Annotated Refseq genes proximal to the lead SNP are shown by position above the X axis. Figure S4. Locus-Zoom plot of the association between MCH (A) and MCV (B) and rs145548796 in the total MEGA study population. Each point represents one SNP; x-axis: increasing position on chromosome 6 left to right; y-axis: -log10(p-value) of the association with MCH SNP correlation with the lead SNP (r2) is colored according to the legend in Figure S4A. Annotated Refseq genes proximal to the lead SNP are shown by position above the X axis. [file 12864_2020_6626_MOESM1_ESM.docx]
